# M. Charriere on Gilding of Surgical Instruments

**Published:** 1842-08-27

**Authors:** 


*On Gilding of Surgical Instruments.* By M. Charriere.—The electrotype has been applied to this purpose in Paris. A letter from M. Charriere was read at the Institute on the 21st of March, in which he says, "Having gilded by M. de Rustz's process a considerable number of surgical instruments and pieces of cutlery, I have submitted them to experiments which seem to me to merit attention. The cutting instruments which I have repeatedly tested on the dead body, have suffered no damage either in the quality of their edge or in their gilding; and the instruments for pressing have preserved all their power of resistance. I have moreover obtained a positive proof that the instruments thus gilded are not subject to rust; and this is an advantage of which the importance may be easily understood, especially for instruments which are intended to remain for some time in the body. I may add that the silver and the platinum plating, applied in the same manner, afford the same results as the gilding."—*Ibid, from Gazette des Hôpitaux.* Mars 24, 1842.

